# From Consultation to Application: Practical Solutions for Improving Maternal and Neonatal Outcomes for Adolescent Aboriginal Mothers at a Local Level

**DOI:** 10.3390/healthcare4040090

**Published:** 2016-12-06

**Authors:** Tracy Reibel, Paula Wyndow, Roz Walker

**Affiliations:** Telethon Kids Institute, University of Western Australia, Nedlands 6009, Australia; Paula.Wyndow@telethonkids.org.au (P.W.); Roz.Walker@telethonkids.org.au (R.W.)

**Keywords:** adolescent pregnancy, Aboriginal women, psychosocial risk, social determinants of health

## Abstract

Adolescent pregnancy has been typically linked to a range of adverse outcomes for mother and child. In Australia, Aboriginal and Torres Strait Islander women have a higher proportion of adolescent births compared with other adolescent Australian women, and are at greater risk of poorer psychosocial and clinical outcomes if they are not well supported during pregnancy and beyond. Drawing on existing literature and consultations with young Aboriginal women and health professionals supporting pregnant Aboriginal women in Western Australia, this paper discusses the importance of creating models of antenatal care using a “social determinants of health” framework. Destigmatizing young parenthood and providing continuity of caregiver in culturally safe services, with culturally competent health professionals provides a means to encourage engagement with the health system and improve health outcomes for young mothers and their babies.

## 1. Introduction

Whilst adolescent pregnancy has been typically linked to adverse birth outcomes such as preterm birth, low birth weight, stillbirth [1,2,3,4], and poorer maternal psychosocial outcomes [5], other studies posit that it is not maternal age nor pregnancy itself that is a direct cause of these adverse outcomes. Rather, it is the pre-existing social determinants of health that are often associated with young parenthood that put adolescent mothers at both psychosocial and clinical risk [6]. These social factors include limited life options as a result of limited employment opportunities, educational attainment and/or poverty and social exclusion [7,8], family disruption, and family and domestic violence [9,10].

However, despite these risk factors, there is also evidence that many adolescent mothers have healthy babies and adjust well to early parenting, particularly when they have strong family and community support and access to high quality maternity health and social services they are confident to attend [11,12,13]. Recent studies have shown that for some young women motherhood has the potential to be transformative [14,15], giving them a sense of purpose in their lives, and preventing them from heading down the “wrong track” (p. 713) [16] . However, the present discourse and social construction of adolescents as “at risk” or vulnerable paints a negative picture of young motherhood, leaving young women feeling like they are under constant surveillance [17]. This may result in young women not coming forward early in their pregnancy to access appropriate antenatal and other social supports for fear of being judged or having their mothering capabilities questioned.

In order to improve health outcomes for adolescent mothers and their babies, health care providers need to be aware of the broader social, environmental, economic and cultural conditions in which women live. A conceptual framework developed by Osborne, Baum and Brown (2013) [18] presents the processes and impacts of colonization as the overarching driver of these social determinants of health for Aboriginal women. Housing, income, education and employment influence, and are influenced by, health behaviors and psychosocial factors, which in turn affect health outcomes. For example, maternal stress, alcohol use and smoking in pregnancy are all associated with poorer health outcomes, including low birth weight babies, preterm and stillbirth [19,20,21] and may potentially be mitigated through improved social conditions.

This article describes how recommendations from two projects might be applied in real world health care settings to improve adolescent maternal and infant outcomes. The projects had sought to understand, firstly, how, why and in what circumstances young Aboriginal women living in Western Australia (WA) access antenatal care; and secondly, how the maternity patient journey might be better managed for Aboriginal women wherever they lived in WA. Note that Aboriginal and Torres Strait Islander peoples refers to the diverse populations which exist across the Australian landmass and islands. As this paper mainly discusses the WA context, the term Aboriginal is used. The authors acknowledge Torres Strait Islander people live and work in WA. Both projects were unequivocal in identifying that in order to improve all pregnant Aboriginal women’s maternal experiences and infant outcomes [22,23,24,25], culturally competent [26,27,28] health professionals providing continuity of care [29,30], and coordinated, collaborative and documented maternity journey planning within and across health services and regions is essential.

Cultural competence is noted as being present when “organizations (which) have a defined set of values and principles, and demonstrate behaviors, attitudes, policies and structures that enable them to work effectively cross-culturally. Cultural competence is a developmental process that evolves over an extended period. Both individuals and organizations are at various levels of awareness, knowledge and skills along the cultural competence continuum” (p. 34) [31]. With respect to continuity of care, this term is used here to describe the provision of continuous care across the maternity pathway by a known carer (usually a midwife) which has been demonstrated to have a beneficial impact on outcomes. Continuity of care enables women to develop a relationship with the same caregiver(s) throughout pregnancy, birth and the postnatal period [32].

The combined project findings were presented to community representatives, maternity care providers and health service management staff at a workshop held to consider how the recommendations might be applied. The purpose was to ask participants to identify practical strategies which they considered could be easily implemented within existing health service capacity in either individual health services or health regions to address issues arising from providing health care to vulnerable young women with complex needs in geographically diverse locations in WA.

### 1.1. Setting the Scene

#### 1.1.1. Teen Pregnancy in Western Australia

In Western Australia, over the past thirty years, the proportion of adolescents (under 20 years) giving birth has fallen from 8.2 percent in 1980 to 3.1 percent in 2014 [33]. However, Aboriginal adolescents are overrepresented in this group, with 18 percent of Aboriginal mothers being under 20 years of age, compared with 3.5 percent of other adolescents. The median age of Aboriginal and Torres Strait Islander women who registered a birth in 2014 was 25.1 years, approximately six years lower than the median age of all WA mothers (30.9 years) [33]. Aboriginal adolescent mothers in WA are also more likely to be living in rural WA and about 14 times as likely to live in remote and very remote areas (23% compared with 1.7%), and 2.5 times as likely to live in the lowest socioeconomic status (SES) areas [34].

A recent longitudinal study of Western Australian mothers showed that between 1998 and 2010, Aboriginal mothers were at an increased risk for both stillbirth and neonatal deaths [35], with Aboriginal adolescent mothers having a significantly increased risk of perinatal loss compared with other adolescent mothers [36]. Aboriginal mothers are also significantly more likely to die at an early age from external causes such as accidents, suicide and homicide than other women, and they tend to be younger with more children [37]. Socioeconomic disadvantage and the pressures of young parenting have been proposed as potential risk factors for these outcomes [38]. The lack of access to home pregnancy test kits, emergency contraception and abortion have also been identified as barriers to young women having autonomy and reproductive control over their fertility [39].

#### 1.1.2. Context of the Geographical Environment on Access to Health

The State of Western Australia comprises over one third (some 2.6 million square kilometers) of the landmass of Australia. The northwest and central areas of WA include some of the most remote and sparsely populated areas in the world. While more than two thirds of the WA population resides in the metropolitan area in the southern part of the state, only one third of the Aboriginal population are located there [40].

**Figure 1:**
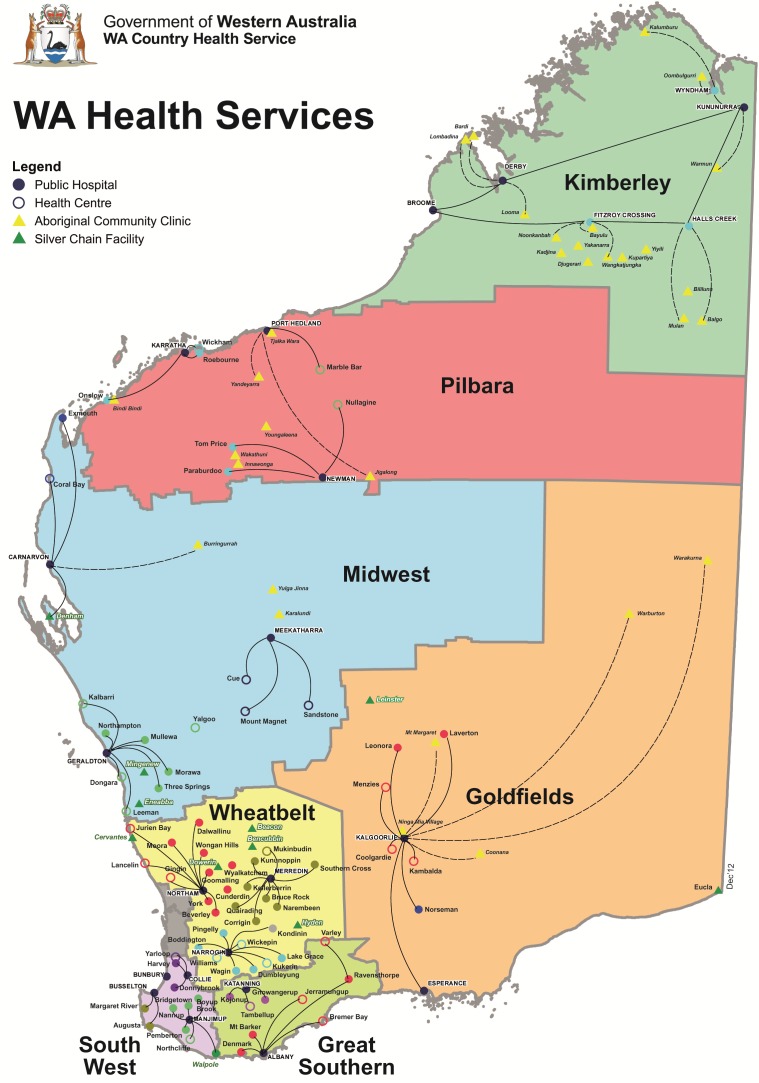
Western Australia health regions [41].

The WA Government’s Department of Health delivers health care through a system of eight health regions (from the Kimberley in the north to the Great Southern in the south), each of which maintains hospitals with varying acuity capability, and, community based health services. (See Figure 1 below.) Additionally, Aboriginal Community Controlled Health Services (ACCHS) or Aboriginal Medical Services (AMS) are in all but one health region, providing culturally safe and targeted health service delivery to local Aboriginal populations.

As an example, the Kimberley region in the far north of WA has the highest proportion of Aboriginal people comparable to other Australians (43% of the overall regional population compared with being 3.5% of the broader WA population). The region also has a higher level of disadvantage, with very low Socio-Economic Indexes for Areas (SEIFA) scores, and more low income and single parent families and unemployed people compared to other regions. The largest population centers in the Kimberley region are some 2200 and 2500 km respectively from the State’s capital, Perth, where the only women’s tertiary hospital is located. Across the Kimberley region, people travelling distances of up to 600 km to reach health services is not uncommon. For pregnant women living in a remote Kimberley community, access to routine pregnancy services such as morphology scans may require being away from home for up to three days. Women with normal risk pregnancies are required to attend a regional center for childbirth, and are relocated from their communities around 36 weeks of pregnancy. If complications are present or arise in pregnancy prior to this time, women may be transferred to Perth, or a regional center. Similar issues to those experienced in the Kimberley region of WA are replicated to varying degrees in other regions of the State.

Therefore, WA presents a number of geographically determined challenges in maintaining both the delivery of, and access to, health care services, often necessitating people traveling substantial distances and being away from home and family for extensive periods of time for routine and emergency health care. In this context, the delivery of maternity services to young Aboriginal women in WA is a vexed issue. This situation requires health care professionals working outside the metropolitan area to consider the environment in which the maternity service is located and how the service is able to deliver culturally safe and high quality maternity care using locally available resources and, when services in other locations are required, how this will be managed to the benefit of young Aboriginal women. Cultural safety has been defined by the Congress of Aboriginal and Torres Strait Islander Nurses and Midwives (2013) as being Aboriginal and Torres Strait Islander specific, and premised on the key role of Aboriginal and Torres Strait Islander communities in determining their health care, including respect for and advancement of the inherent rights of Aboriginal and Torres Strait Islander peoples [42].

#### 1.1.3. Context of Adolescent Pregnancy

##### Why Teenage Pregnancy Occurs?

Some young people choose early parenthood for personal, cultural or familial reasons [43]. For example, the influence of early family formation and caregiving [44], and the representation of motherhood as a rite of passage to adulthood [45,46] may signify to young women that choosing motherhood is a desirable and valued life choice. Additionally, young women may view early pregnancy as having the potential to be transformative [14].

In contrast, when pregnancy is unplanned, it is typically influenced by limited education and employment opportunities [8], early sexual activity [47], physical or sexual abuse, coercion [48], and scant sexual and reproductive knowledge [49]. These factors put young women at risk for a range of psychosocial risk factors, as they manage the competing demands of becoming a parent while dealing with their own developmental needs [50]. The increased likelihood of psychosocial risks in this cohort, such as homelessness, underemployment and unemployment, lack of familial support and/or family dysfunction, disengagement from educational opportunities, lack of knowledge/awareness of support services, all have the potential to contribute to poor clinical outcomes for adolescent mothers if these aspects of a young woman’s life are not taken into account when health professionals are planning her maternity care [51].

##### Supporting Adolescents Regardless of Causal Factors

Regardless of the circumstances in which young Aboriginal women find themselves pregnant, if they decide to proceed with the pregnancy, understanding the factors which encourage them to engage in antenatal care is crucial to improving their access to, and uptake of, maternity services. Rather than stigmatizing young mothers and risking their disengagement from health services [45], linking them with social support services to assist them to transition to motherhood and access what they need for themselves to provide a nurturing and safe/secure environment in which to raise their child is critical. Such an approach includes reducing the likelihood of a rapid second pregnancy, as this is more likely than a first birth in adolescence to lead into a life of poverty and deprivation [37].

## 2. Method

In the period 2012–2014, two projects were conducted by Telethon Kids Institute in partnership with WA Department of Health’s Aboriginal Maternity Services Support Unit. The purpose of the projects was to: (1) examine the pregnancy journey for young Aboriginal women in WA; and, (2) determine the feasibility of an integrated model of care for Aboriginal women in an attempt to address known health systems issues.

(1) The Young Aboriginal Women’s Voices on Pregnancy Care project was conducted as a descriptive qualitative research study with data collection undertaken using a culturally sensitive, semi-structured interview schedule incorporating a personal and conversational manner known as yarning [52]. Data was analysed using standard qualitative analysis techniques, identifying themes and sub-topics. A total of 84 individual or group interviews were conducted with 28 women in the target group (16–21 years) and 56 senior and elder Aboriginal women and non-Aboriginal service providers in locations across WA, including six out of the eight health regions. The field researchers were an Aboriginal cultural consultant highly experienced in working with young Aboriginal mothers in her role as an Aboriginal Health Worker and a non-Aboriginal researcher with extensive experience in conducting interviews with Aboriginal people. Study approval was granted by the Western Australian Aboriginal Health Ethics Committee (WAAHEC) (2013:443) and the Western Australian Country Health Service (WACHS) (2013:05), Women’s and Newborns Health Service (2013-038EW), and North and South Metropolitan Health Services Human Research Ethics Committees (HREC) (reciprocal approval). Research approval was also granted by the Kimberley Aboriginal Health Planning Forum Research Sub-Committee.
(2) The Aboriginal Women’s Patient Journeys project was a feasibility study to determine what an integrated model of care for Aboriginal women in WA during pregnancy might include. Over 140 health professionals from 53 different organizations across WA participated in either focus groups or individual interviews. To identify themes, each focus group or interview printed record and taped transcript was analysed and common themes and topics were then translated into a matrix to allow comparison across regions. These matrices were reviewed by the research team for confirmation of content and to conclude what similarities and contrasts were apparent across regions. The data was analysed according to standard qualitative data analysis protocols. Ethics approval for this project was obtained from WAAHEC (2013:500) and WACHS HREC (2013:21). The King Edward Memorial Hospital HREC committee assessed the project as a quality improvement project, thus it was entered into Governance Evidence Knowledge Outcomes (GEKO) Safety and Quality database as a quality audit. Express written permission was obtained from the Chief Executive Officer or Board for every ACCHS or AMS involved.

In line with literature referred to earlier and other previous research [53], the findings of both projects showed that prioritizing models of antenatal care which are designed to promote respect for young women’s choices, are culturally safe, and are provided by culturally competent health professionals are the most likely conditions that will encourage young women to attend health services for antenatal care early in pregnancy. When this approach includes collaborative arrangements with local social support services, such as housing, income support, and social and emotional wellbeing and/or parenting programs, a practical means to reduce psychosocial risk exists. By supporting a young woman to have her basic needs met, for example, to live in secure housing and obtain adequate and appropriate nutrition, this in turn leads to more opportunity to engage with a young woman to address her clinical needs. By creating the means for frequent contact between young women and their maternity care providers, in environments where they feel most comfortable, such as at an ACCHS or in their own home where this is acceptable, the opportunity to both educate young women and monitor their clinical wellbeing, and to mitigate risks as these as arise, promotes the best circumstances for improved maternal and neonatal perinatal outcomes.

### 2.1. Applying the Research Findings

At the end of 2014, the recommendations from both project reports were presented at a workshop of about 80 Aboriginal and other representatives including health professionals, maternity service managers, and community representatives. While some recommendations from the project reports related to health system changes, requiring policy changes and reallocation of resources to create more effective fit-for-purpose service delivery mechanisms, other recommendations could potentially be implemented by existing local or regional health services within available resources. The recommendations were posed to workshop participants for discussion with the recommendations specific to Aboriginal adolescents summarized here:

- Young Aboriginal women need pregnancy care that supports them during a vulnerable time in their lives and respects their choice, privacy and confidentiality at all times and in all service settings.
- Aboriginal family relationships are crucial to encouraging early and ongoing antenatal engagement by young Aboriginal women. Services need to build relationships with influential local women Elders/community members to support knowledge exchange of pregnancy and childbirth and local cultural practices; dissemination of information about pregnancy care and available services throughout communities; and assistance with identification of young pregnant women to encourage engagement with health care.
- Dedicated spaces are required where women can come together safely to “yarn” (talk).
- Culturally safe models of care (with continuity of carer) provided by culturally competent health professionals and other health service staff are needed to maintain relationships with young pregnant women.
- Adolescents moving between communities were viewed as being most at risk of not accessing antenatal care. Women who need to relocate to give birth are at risk of falling through service gaps and having negative pregnancy and birthing experiences as a result.
- A documented patient relocation pathway is required to support young Aboriginal women to feel safe. This includes securing appropriate transport from and back to home communities; assistance for escort/support people to be present for the duration of the pregnant woman’s relocation (including changes of support people over that time); accommodation options and social support services at the relocation site, including access to an Aboriginal Liaison Officer/Community Care or Health Worker; detailed information about the cultural practices/expectations of individual Aboriginal women forwarded by the home community health service staff for the information of health service staff at the relocation site.

### 2.2. Identifying Actions for Implementation

The workshop was an opportunity for participants to identify what actions might be undertaken within participants own service delivery parameters to address known issues associated with both the early engagement of young Aboriginal women in their maternity care and with their journey planning. The purpose of the exercise was for participants to identify and highlight what could be achieved within known existing resources and structures by taking account of the variety of conditions in which services were provided, including the availability of other services and supports.

The key focus of the discussion was: what is required for young pregnant women to access a range of routine maternity services, some of which may potentially be located in different places not necessarily convenient to a usual place of residence? Considerations included: the types of transport that may, or may not be, available within a location; and, what is involved in accessing the service (whether it is a mainstream service or ACCHS/AMS), and how welcoming (culturally safe) the service is for young women? Additionally, the participants considered how a young woman might be able to manage attending different clinical services such as blood tests, morphology scans, or glucose tolerance tests from her home location, and what possibility there is for these to be coordinated wherever possible?

Participants worked in groups to brainstorm specific questions associated with the projects’ recommendations, with a focus on what was realistically achievable. Actions that may be able to be undertaken were recorded by each group in response to each question. These actions were then presented to the whole gathering of participants, with further facilitated discussion regarding the feasibility and means of achieving the actions within current service delivery parameters.

The participant information collected during the session was later analysed and summarized before being provided back to the Aboriginal Maternity Services Support Unit for their use to advocate for changes in the way maternity care is provided, taking into account local context and need. Specific strategies relating to adolescent mothers are summarized in Table 1.

## 3. Discussion

This paper provides an overview of the existing literature and the strategies that arose from consultations with stakeholders about how to better engage and support adolescent Aboriginal women in pregnancy. The first project research outcomes focused on gathering evidence related to the needs of young Aboriginal women during pregnancy, and the issues present in the unique context of provision of maternity care in WA. The research was intended to inform those working in maternity care with knowledge about what young women want, expect and need from health professionals during a pregnancy. The second project outcomes identified the myriad of issues associated with providing maternity care to Aboriginal women residing in geographically diverse locations, what might be addressed at a local service or regional level and what issues required a more high level response from the WA Department of Health.

The strategies identified by the stakeholders in the workshop were focused on ways in which changes in the provision of antenatal care and patient journey planning for young Aboriginal women may be achieved in different local service delivery environments, taking into account a range of cultural and geographical challenges. The provision of culturally safe health services which offer trusting and respectful relationships between adolescent pregnant women and health care providers in their local or home community was identified as important to establish the means to effectively engage with young pregnant women. The discussion and documentation of journey planning in consultation with each young woman was noted as a means to promote understanding of the reality of the circumstances and as much as possible meet the needs of young women within what is currently available. This included both pregnancy and post birth discharge planning, to ensure that young women had adequate follow up for themselves and their infants.

Overall, the outcomes confirmed the importance of service providers undertaking careful planning and delivery of high quality, holistic antenatal care relevant to the local population, with collaborative targeted supports put in place to assist young women having babies. The provision of antenatal care in this context is concerned with taking account of the cultural, environmental, and social conditions in which a young woman is located and planning care in collaboration with all of the support services that are relevant to assisting young women achieve a good outcome for herself and her infant, both during pregnancy and after childbirth. More often than not, this includes the integral role of family members, particularly Elder women. This social determinants of health approach, which looks beyond clinical care and immediate psychosocial risk to firstly consider young women’s place within their community and the available supports (including if their basic needs are being met) is a practical way to encourage and support young women’s transition to parenting.

## 4. Conclusions

Prioritizing the social determinants of health within models of antenatal care is more likely to support good outcomes for adolescent Aboriginal mothers. Destigmatizing young parenthood and providing care in culturally safe services, with culturally competent health professionals contributes to encouraging engagement with the health system which in turn will improve health outcomes for young mothers and their babies. The practical nature of the actions identified in the workshops reflected on the outcomes and recommendations of prior research and may be transferable to other similar environments where the delivery of maternity health care services also includes complex cultural and geographical considerations. Often the solutions with regard to quality health care in complex circumstances are related to the very basis of health care provision, which we contend is based on respectful relationships and communication with the person receiving care and through effective linkage with support services. So the availability of the right carer/s, at the right time, in the right circumstances may alleviate some of the psychosocial risk factors associated with early parenthood and provide a means to address clinical risks as these arise. More so, where the environmental, cultural and social conditions are at the forefront of service delivery, better overall outcomes are likely achievable.

Health professionals should not be deterred by health systems issues, but instead build on what is available locally, and use the strengths in community (particularly community Elders where this is relevant) to support the delivery of health care. Health professionals can also advocate for continuity of carer in the delivery of maternity care to adolescent mothers as a means to improve both maternal and neonatal outcomes.

## Figures and Tables

**Table 1:** Practical strategies to support Aboriginal adolescents during their pregnancy journey.

Pregnancy Topic	Summary of Participant Identified Strategies
**Pre-Pregnancy** Participants were asked what they understood about the local context in which service providers worked and who to speak with to develop knowledge of the community.	Service providers creating opportunities for dialogue with the whole community, commencing with Elder women. Creating innovative approaches to sexual and reproductive health in consultation with Elders and grandmothers in each community. Creating culturally safe physical spaces where women’s business can be discussed. Service providers maintaining communication with other service providers in the community and keeping informed about what each services offers and where collaboration can occur.
**Pregnancy** Participants were asked to consider what was needed to engage with young women early in pregnancy.	Initiating and maintaining a respectful relationship with a young woman throughout her pregnancy through the provision of culturally safe services which address her specific needs and include her significant family members. Being aware of local resources and issues, for example, whether transport is required (to and from antenatal appointments), or if home visiting is appropriate or feasible. The importance of yarning—giving time and respect for a young woman’s story to unfold over time, as trust is built, and for this to be documented sufficiently to avoid repetition of enquiry. Knowing what local supports are available—family, local, State and Federal government, Aboriginal Community Controlled Health Services and other corporations, other philanthropic sources, and how supports can be collaboratively integrated to support a young woman during and after pregnancy.
**Relocation/Preparing to Birth** Participants were asked to consider how they might go about planning relocation in consultation with the young woman.	Address the issue of birth place early in pregnancy and begin preparation with the young woman and her support people about what plans can be put in place when relocation is required in the final weeks of pregnancy. Establishing lines of communication between the woman’s antenatal care provider and intended birth place. What position or department can the home community service provider contact at the hospital for information? Is there an Aboriginal Health Worker, an Aboriginal liaison person, social work department, or other who can be advised about the relocation plans for the young woman? Important to take the time to map, plan and document this journey with and for young women.
**Discharge/Relocation to Home Community** Participants were asked to consider how they might go about planning and documenting a discharge for a young women for her return to the community.	Establishing and maintaining lines of communication between the hospital and home community health services, including the Aboriginal Community Controlled Health Service, Child Health, or other relevant health service in the woman’s home community. Discharge should include asking each woman where she intends going after discharge from hospital as it may not immediately be to the woman’s home community. In this event, sending discharge forms to several places that a woman identifies she might go promotes greater likelihood the woman can have follow up maternal and infant care relevant to their needs. In the local service network stress the importance of postnatal teams to support women—e.g., Child Health Nurse and Aboriginal Health/Community Care Worker or grandmother, with flexibility to go to the woman’s home.
